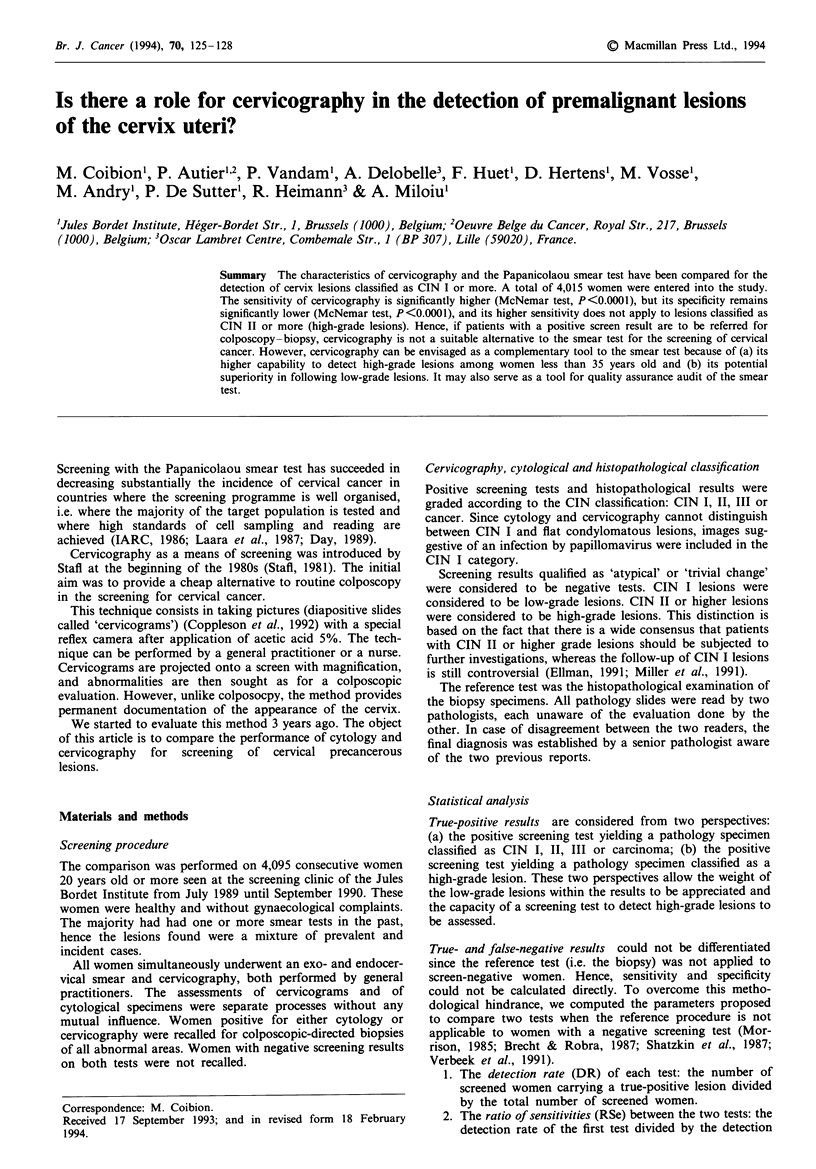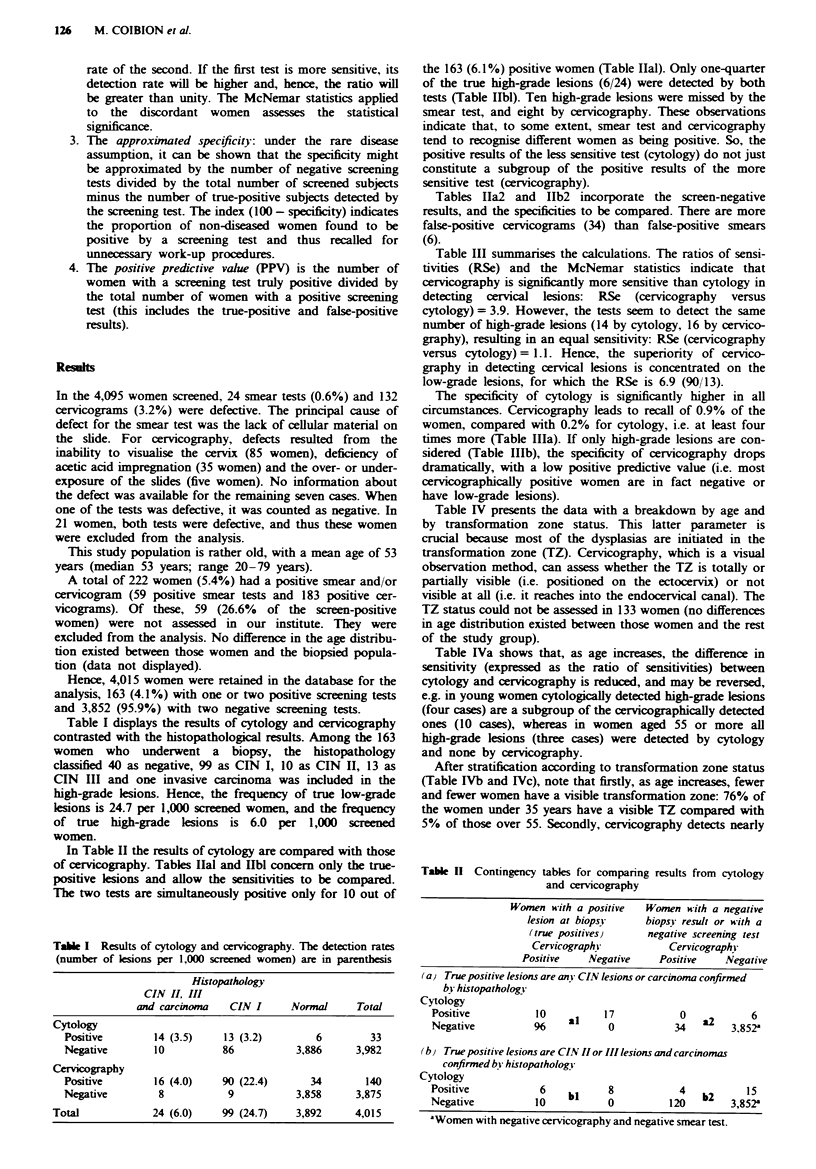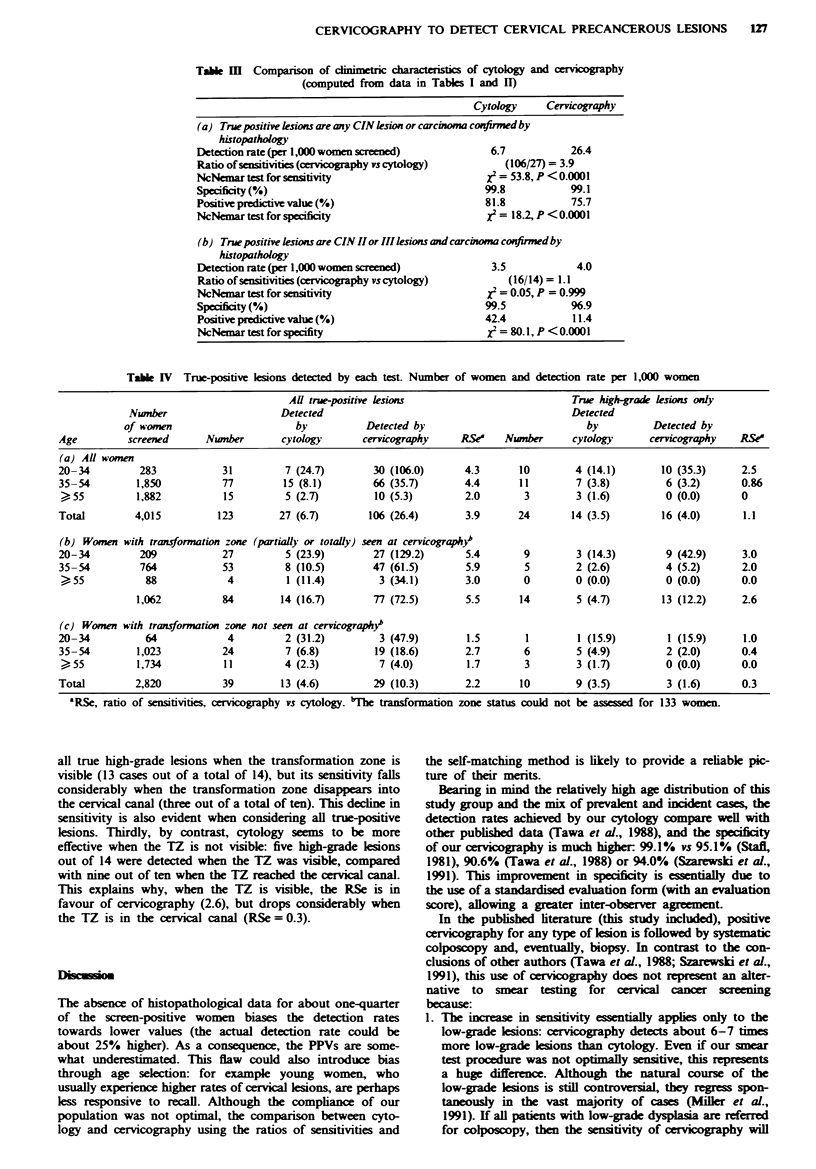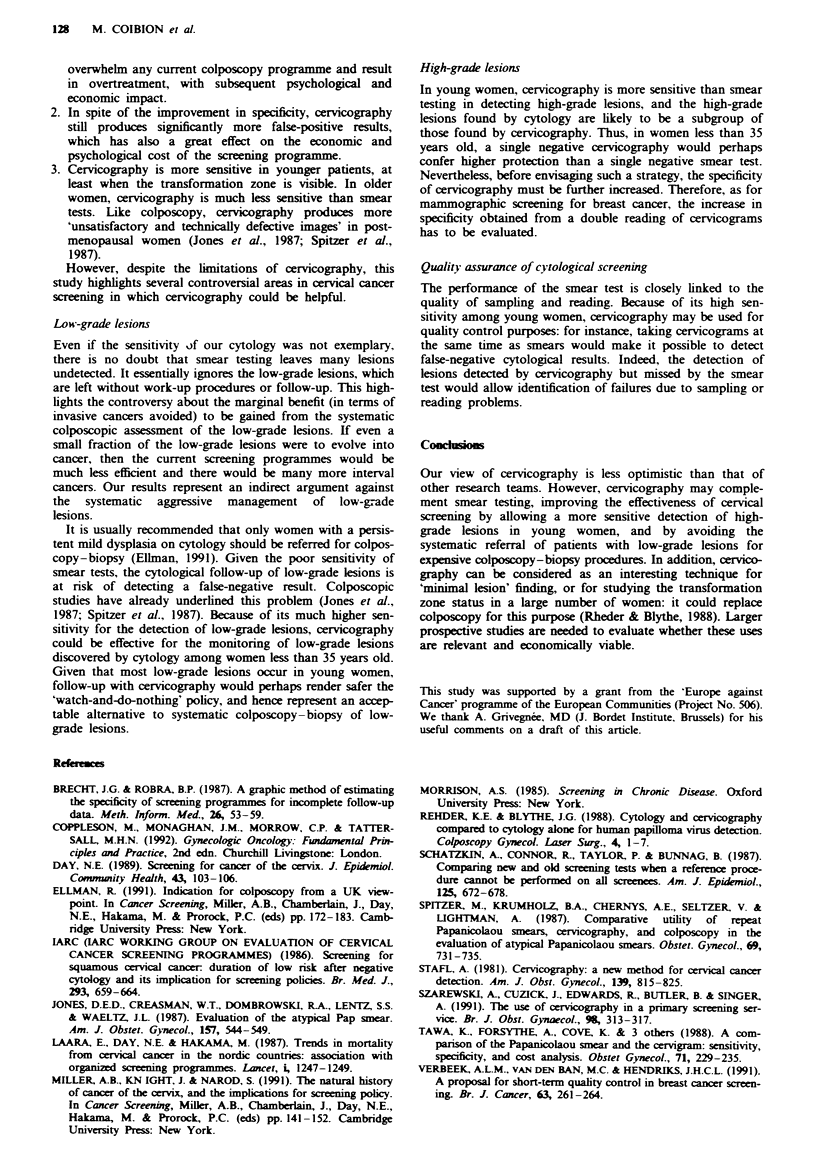# Is there a role for cervicography in the detection of premalignant lesions of the cervix uteri?

**DOI:** 10.1038/bjc.1994.260

**Published:** 1994-07

**Authors:** M. Coibion, P. Autier, P. Vandam, A. Delobelle, F. Huet, D. Hertens, M. Vosse, M. Andry, P. De Sutter, R. Heimann

**Affiliations:** Jules Bordet Institute, Brussels, Belgium.

## Abstract

The characteristics of cervicography and the Papanicolaou smear test have been compared for the detection of cervix lesions classified as CIN I or more. A total of 4,015 women were entered into the study. The sensitivity of cervicography is significantly higher (McNemar test, P < 0.0001), but its specificity remains significantly lower (McNemar test, P < 0.0001), and its higher sensitivity does not apply to lesions classified as CIN II or more (high-grade lesions). Hence, if patients with a positive screen result are to be referred for colposcopy-biopsy, cervicography is not a suitable alternative to the smear test for the screening of cervical cancer. However, cervicography can be envisaged as a complementary tool to the smear test because of (a) its higher capability to detect high-grade lesions among women less than 35 years old and (b) its potential superiority in following low-grade lesions. It may also serve as a tool for quality assurance audit of the smear test.


Br. J. Cancer (1994), 70, 125-128 © Macmillan Press Ltd., 1994

### Is there a role for cervicography in the detection of premalignant lesions of the cervix uteri?

M. Coibion[1], P. Autier[1,2], P. Vandam[1], A. Delobelle[3], F. Huet[1], D. Hertens[1], M. Vosse[1], M. Andry[1], P. De Sutter[1], R. Heimann[3] & A. Miloiu[1]

[1]*Jules Bordet Institute, Héger-Bordet Str., 1, Brussels (1000), Belgium;* [2]*Oeuvre Belge du Cancer, Royal Str., 217, Brussels (1000), Belgium;* [3]*Oscar Lambret Centre, Combemale Str., 1 (BP 307), Lille (59020), France.*

**Summary** The characteristics of cervicography and the Papanicolaou smear test have been compared for the detection of cervix lesions classified as CIN I or more. A total of 4,015 women were entered into the study. The sensitivity of cervicography is significantly higher (McNemar test, $P<0.0001$), but its specificity remains significantly lower (McNemar test, $P<0.0001$), and its higher sensitivity does not apply to lesions classified as CIN II or more (high-grade lesions). Hence, if patients with a positive screen result are to be referred for colposcopy-biopsy, cervicography is not a suitable alternative to the smear test for the screening of cervical cancer. However, cervicography can be envisaged as a complementary tool to the smear test because of (a) its higher capability to detect high-grade lesions among women less than 35 years old and (b) its potential superiority in following low-grade lesions. It may also serve as a tool for quality assurance audit of the smear test.

Screening with the Papanicolaou smear test has succeeded in decreasing substantially the incidence of cervical cancer in countries where the screening programme is well organised, i.e. where the majority of the target population is tested and where high standards of cell sampling and reading are achieved (IARC, 1986; Laara *et al.*, 1987; Day, 1989).

Cervicography as a means of screening was introduced by Stafl at the beginning of the 1980s (Stafl, 1981). The initial aim was to provide a cheap alternative to routine colposcopy in the screening for cervical cancer.

This technique consists in taking pictures (diapositive slides called 'cervicograms') (Coppleson *et al.*, 1992) with a special reflex camera after application of acetic acid 5%. The technique can be performed by a general practitioner or a nurse. Cervicograms are projected onto a screen with magnification, and abnormalities are then sought as for a colposcopic evaluation. However, unlike colposocpy, the method provides permanent documentation of the appearance of the cervix.

We started to evaluate this method 3 years ago. The object of this article is to compare the performance of cytology and cervicography for screening of cervical precancerous lesions.

### Materials and methods

### *Screening procedure*

The comparison was performed on 4,095 consecutive women 20 years old or more seen at the screening clinic of the Jules Bordet Institute from July 1989 until September 1990. These women were healthy and without gynaecological complaints. The majority had had one or more smear tests in the past, hence the lesions found were a mixture of prevalent and incident cases.

All women simultaneously underwent an exo- and endocervical smear and cervicography, both performed by general practitioners. The assessments of cervicograms and of cytological specimens were separate processes without any mutual influence. Women positive for either cytology or cervicography were recalled for colposcopic-directed biopsies of all abnormal areas. Women with negative screening results on both tests were not recalled.

Correspondence: M. Coibion.
Received 17 September 1993; and in revised form 18 February 1994.

### *Cervicography, cytological and histopathological classification*

Positive screening tests and histopathological results were graded according to the CIN classification: CIN I, II, III or cancer. Since cytology and cervicography cannot distinguish between CIN I and flat condylomatous lesions, images suggestive of an infection by papillomavirus were included in the CIN I category.

Screening results qualified as 'atypical' or 'trivial change' were considered to be negative tests. CIN I lesions were considered to be low-grade lesions. CIN II or higher lesions were considered to be high-grade lesions. This distinction is based on the fact that there is a wide consensus that patients with CIN II or higher grade lesions should be subjected to further investigations, whereas the follow-up of CIN I lesions is still controversial (Ellman, 1991; Miller *et al.*, 1991).

The reference test was the histopathological examination of the biopsy specimens. All pathology slides were read by two pathologists, each unaware of the evaluation done by the other. In case of disagreement between the two readers, the final diagnosis was established by a senior pathologist aware of the two previous reports.

### *Statistical analysis*

*True-positive results* are considered from two perspectives: (a) the positive screening test yielding a pathology specimen classified as CIN I, II, III or carcinoma; (b) the positive screening test yielding a pathology specimen classified as a high-grade lesion. These two perspectives allow the weight of the low-grade lesions within the results to be appreciated and the capacity of a screening test to detect high-grade lesions to be assessed.

*True- and false-negative results* could not be differentiated since the reference test (i.e. the biopsy) was not applied to screen-negative women. Hence, sensitivity and specificity could not be calculated directly. To overcome this methodological hindrance, we computed the parameters proposed to compare two tests when the reference procedure is not applicable to women with a negative screening test (Morrison, 1985; Brecht & Robra, 1987; Shatzkin *et al.*, 1987; Verbeek *et al.*, 1991).

1. The *detection rate* (DR) of each test: the number of screened women carrying a true-positive lesion divided by the total number of screened women.
2. The *ratio of sensitivities* (RSe) between the two tests: the detection rate of the first test divided by the detection rate of the second. If the first test is more sensitive, its detection rate will be higher and, hence, the ratio will be greater than unity. The McNemar statistics applied to the discordant women assesses the statistical significance.

3. The *approximated specificity*: under the rare disease assumption, it can be shown that the specificity might be approximated by the number of negative screening tests divided by the total number of screened subjects minus the number of true-positive subjects detected by the screening test. The index (100 – specificity) indicates the proportion of non-diseased women found to be positive by a screening test and thus recalled for unnecessary work-up procedures.
4. The *positive predictive value* (PPV) is the number of women with a screening test truly positive divided by the total number of women with a positive screening test (this includes the true-positive and false-positive results).

### Results

In the 4,095 women screened, 24 smear tests (0.6%) and 132 cervicograms (3.2%) were defective. The principal cause of defect for the smear test was the lack of cellular material on the slide. For cervicography, defects resulted from the inability to visualise the cervix (85 women), deficiency of acetic acid impregnation (35 women) and the over- or under-exposure of the slides (five women). No information about the defect was available for the remaining seven cases. When one of the tests was defective, it was counted as negative. In 21 women, both tests were defective, and thus these women were excluded from the analysis.

This study population is rather old, with a mean age of 53 years (median 53 years; range 20–79 years).

A total of 222 women (5.4%) had a positive smear and/or cervicogram (59 positive smear tests and 183 positive cervicograms). Of these, 59 (26.6% of the screen-positive women) were not assessed in our institute. They were excluded from the analysis. No difference in the age distribution existed between those women and the biopsied population (data not displayed).

Hence, 4,015 women were retained in the database for the analysis, 163 (4.1%) with one or two positive screening tests and 3,852 (95.9%) with two negative screening tests.

Table I displays the results of cytology and cervicography contrasted with the histopathological results. Among the 163 women who underwent a biopsy, the histopathology classified 40 as negative, 99 as CIN I, 10 as CIN II, 13 as CIN III and one invasive carcinoma was included in the high-grade lesions. Hence, the frequency of true low-grade lesions is 24.7 per 1,000 screened women, and the frequency of true high-grade lesions is 6.0 per 1,000 screened women.

In Table II the results of cytology are compared with those of cervicography. Tables IIal and IIbl concern only the true-positive lesions and allow the sensitivities to be compared. The two tests are simultaneously positive only for 10 out of the 163 (6.1%) positive women (Table IIal). Only one-quarter of the true high-grade lesions (6/24) were detected by both tests (Table IIbl). Ten high-grade lesions were missed by the smear test, and eight by cervicography. These observations indicate that, to some extent, smear test and cervicography tend to recognise different women as being positive. So, the positive results of the less sensitive test (cytology) do not just constitute a subgroup of the positive results of the more sensitive test (cervicography).

Tables IIa2 and IIb2 incorporate the screen-negative results, and the specificities to be compared. There are more false-positive cervicograms (34) than false-positive smears (6).

Table III summarises the calculations. The ratios of sensitivities (RSe) and the McNemar statistics indicate that cervicography is significantly more sensitive than cytology in detecting cervical lesions: RSe (cervicography versus cytology) = 3.9. However, the tests seem to detect the same number of high-grade lesions (14 by cytology, 16 by cervicography), resulting in an equal sensitivity: RSe (cervicography versus cytology) = 1.1. Hence, the superiority of cervicography in detecting cervical lesions is concentrated on the low-grade lesions, for which the RSe is 6.9 (90/13).

The specificity of cytology is significantly higher in all circumstances. Cervicography leads to recall of 0.9% of the women, compared with 0.2% for cytology, i.e. at least four times more (Table IIIa). If only high-grade lesions are considered (Table IIIb), the specificity of cervicography drops dramatically, with a low positive predictive value (i.e. most cervicographically positive women are in fact negative or have low-grade lesions).

Table IV presents the data with a breakdown by age and by transformation zone status. This latter parameter is crucial because most of the dysplasias are initiated in the transformation zone (TZ). Cervicography, which is a visual observation method, can assess whether the TZ is totally or partially visible (i.e. positioned on the ectocervix) or not visible at all (i.e. it reaches into the endocervical canal). The TZ status could not be assessed in 133 women (no differences in age distribution existed between those women and the rest of the study group).

Table IVa shows that, as age increases, the difference in sensitivity (expressed as the ratio of sensitivities) between cytology and cervicography is reduced, and may be reversed, e.g. in young women cytologically detected high-grade lesions (four cases) are a subgroup of the cervicographically detected ones (10 cases), whereas in women aged 55 or more all high-grade lesions (three cases) were detected by cytology and none by cervicography.

After stratification according to transformation zone status (Table IVb and IVc), note that firstly, as age increases, fewer and fewer women have a visible transformation zone: 76% of the women under 35 years have a visible TZ compared with 5% of those over 55. Secondly, cervicography detects nearly

**Table I** Results of cytology and cervicography. The detection rates (number of lesions per 1,000 screened women) are in parenthesis

| | *Histopathology* CIN II, III and carcinoma | CIN I | Normal | Total |
|---|---|---|---|---|
| Cytology | | | | |
| Positive | 14 (3.5) | 13 (3.2) | 6 | 33 |
| Negative | 10 | 86 | 3,886 | 3,982 |
| Cervicography | | | | |
| Positive | 16 (4.0) | 90 (22.4) | 34 | 140 |
| Negative | 8 | 9 | 3,858 | 3,875 |
| Total | 24 (6.0) | 99 (24.7) | 3,892 | 4,015 |

**Table II** Contingency tables for comparing results from cytology and cervicography

| | *Women with a positive lesion at biopsy (true positives)* *Cervicography* Positive | | Negative | *Women with a negative biopsy result or with a negative screening test* *Cervicography* Positive | | Negative |
|---|---|---|---|---|---|---|
| *(a) True positive lesions are any CIN lesions or carcinoma confirmed by histopathology* | | | | | | |
| Cytology | | | | | | |
| Positive | 10 | a1 | 17 | 0 | a2 | 6 |
| Negative | 96 | | 0 | 34 | | 3,852[a] |
| *(b) True positive lesions are CIN II or III lesions and carcinomas confirmed by histopathology* | | | | | | |
| Cytology | | | | | | |
| Positive | 6 | b1 | 8 | 4 | b2 | 15 |
| Negative | 10 | | 0 | 120 | | 3,852[a] |

[a] Women with negative cervicography and negative smear test.

**Table III** Comparison of clinimetric characteristics of cytology and cervicography (computed from data in Tables I and II)

| | *Cytology* | *Cervicography* |
|---|---|---|
| *(a) True positive lesions are any CIN lesion or carcinoma confirmed by histopathology* | | |
| Detection rate (per 1,000 women screened) | 6.7 | 26.4 |
| Ratio of sensitivities (cervicography *vs* cytology) | (106/27) = 3.9 | |
| NcNemar test for sensitivity | $\chi^2 = 53.8, P < 0.0001$ | |
| Specificity (%) | 99.8 | 99.1 |
| Positive predictive value (%) | 81.8 | 75.7 |
| NcNemar test for specificity | $\chi^2 = 18.2, P < 0.0001$ | |
| *(b) True positive lesions are CIN II or III lesions and carcinoma confirmed by histopathology* | | |
| Detection rate (per 1,000 women screened) | 3.5 | 4.0 |
| Ratio of sensitivities (cervicography *vs* cytology) | (16/14) = 1.1 | |
| NcNemar test for sensitivity | $\chi^2 = 0.05, P = 0.999$ | |
| Specificity (%) | 99.5 | 96.9 |
| Positive predictive value (%) | 42.4 | 11.4 |
| NcNemar test for specifity | $\chi^2 = 80.1, P < 0.0001$ | |

**Table IV** True-positive lesions detected by each test. Number of women and detection rate per 1,000 women

| | | *All true-positive lesions* | | | | *True high-grade lesions only* | | | |
|---|---|---|---|---|---|---|---|---|---|
| *Age* | *Number of women screened* | *Number* | *Detected by cytology* | *Detected by cervicography* | *RSe*[a] | *Number* | *Detected by cytology* | *Detected by cervicography* | *RSe*[a] |
| *(a) All women* | | | | | | | | | |
| 20–34 | 283 | 31 | 7 (24.7) | 30 (106.0) | 4.3 | 10 | 4 (14.1) | 10 (35.3) | 2.5 |
| 35–54 | 1,850 | 77 | 15 (8.1) | 66 (35.7) | 4.4 | 11 | 7 (3.8) | 6 (3.2) | 0.86 |
| ⩾55 | 1,882 | 15 | 5 (2.7) | 10 (5.3) | 2.0 | 3 | 3 (1.6) | 0 (0.0) | 0 |
| Total | 4,015 | 123 | 27 (6.7) | 106 (26.4) | 3.9 | 24 | 14 (3.5) | 16 (4.0) | 1.1 |
| *(b) Women with transformation zone (partially or totally) seen at cervicography*[b] | | | | | | | | | |
| 20–34 | 209 | 27 | 5 (23.9) | 27 (129.2) | 5.4 | 9 | 3 (14.3) | 9 (42.9) | 3.0 |
| 35–54 | 764 | 53 | 8 (10.5) | 47 (61.5) | 5.9 | 5 | 2 (2.6) | 4 (5.2) | 2.0 |
| ⩾55 | 88 | 4 | 1 (11.4) | 3 (34.1) | 3.0 | 0 | 0 (0.0) | 0 (0.0) | 0.0 |
| | 1,062 | 84 | 14 (16.7) | 77 (72.5) | 5.5 | 14 | 5 (4.7) | 13 (12.2) | 2.6 |
| *(c) Women with transformation zone not seen at cervicography*[b] | | | | | | | | | |
| 20–34 | 64 | 4 | 2 (31.2) | 3 (47.9) | 1.5 | 1 | 1 (15.9) | 1 (15.9) | 1.0 |
| 35–54 | 1,023 | 24 | 7 (6.8) | 19 (18.6) | 2.7 | 6 | 5 (4.9) | 2 (2.0) | 0.4 |
| ⩾55 | 1,734 | 11 | 4 (2.3) | 7 (4.0) | 1.7 | 3 | 3 (1.7) | 0 (0.0) | 0.0 |
| Total | 2,820 | 39 | 13 (4.6) | 29 (10.3) | 2.2 | 10 | 9 (3.5) | 3 (1.6) | 0.3 |

[a]RSe, ratio of sensitivities, cervicography *vs* cytology. [b]The transformation zone status could not be assessed for 133 women.

all true high-grade lesions when the transformation zone is visible (13 cases out of a total of 14), but its sensitivity falls considerably when the transformation zone disappears into the cervical canal (three out of a total of ten). This decline in sensitivity is also evident when considering all true-positive lesions. Thirdly, by contrast, cytology seems to be more effective when the TZ is not visible: five high-grade lesions out of 14 were detected when the TZ was visible, compared with nine out of ten when the TZ reached the cervical canal. This explains why, when the TZ is visible, the RSe is in favour of cervicography (2.6), but drops considerably when the TZ is in the cervical canal (RSe = 0.3).

### Discussion

The absence of histopathological data for about one-quarter of the screen-positive women biases the detection rates towards lower values (the actual detection rate could be about 25% higher). As a consequence, the PPVs are somewhat underestimated. This flaw could also introduce bias through age selection: for example young women, who usually experience higher rates of cervical lesions, are perhaps less responsive to recall. Although the compliance of our population was not optimal, the comparison between cytology and cervicography using the ratios of sensitivities and the self-matching method is likely to provide a reliable picture of their merits.

Bearing in mind the relatively high age distribution of this study group and the mix of prevalent and incident cases, the detection rates achieved by our cytology compare well with other published data (Tawa *et al.*, 1988), and the specificity of our cervicography is much higher: 99.1% *vs* 95.1% (Stafl, 1981), 90.6% (Tawa *et al.*, 1988) or 94.0% (Szarewski *et al.*, 1991). This improvement in specificity is essentially due to the use of a standardised evaluation form (with an evaluation score), allowing a greater inter-observer agreement.

In the published literature (this study included), positive cervicography for any type of lesion is followed by systematic colposcopy and, eventually, biopsy. In contrast to the conclusions of other authors (Tawa *et al.*, 1988; Szarewski *et al.*, 1991), this use of cervicography does not represent an alternative to smear testing for cervical cancer screening because:

1. The increase in sensitivity essentially applies only to the low-grade lesions: cervicography detects about 6–7 times more low-grade lesions than cytology. Even if our smear test procedure was not optimally sensitive, this represents a huge difference. Although the natural course of the low-grade lesions is still controversial, they regress spontaneously in the vast majority of cases (Miller *et al.*, 1991). If all patients with low-grade dysplasia are referred for colposcopy, then the sensitivity of cervicography will overwhelm any current colposcopy programme and result in overtreatment, with subsequent psychological and economic impact.
2. In spite of the improvement in specificity, cervicography still produces significantly more false-positive results, which has also a great effect on the economic and psychological cost of the screening programme.
3. Cervicography is more sensitive in younger patients, at least when the transformation zone is visible. In older women, cervicography is much less sensitive than smear tests. Like colposcopy, cervicography produces more 'unsatisfactory and technically defective images' in post-menopausal women (Jones et al., 1987; Spitzer et al., 1987).

However, despite the limitations of cervicography, this study highlights several controversial areas in cervical cancer screening in which cervicography could be helpful.

*Low-grade lesions*

Even if the sensitivity of our cytology was not exemplary, there is no doubt that smear testing leaves many lesions undetected. It essentially ignores the low-grade lesions, which are left without work-up procedures or follow-up. This highlights the controversy about the marginal benefit (in terms of invasive cancers avoided) to be gained from the systematic colposcopic assessment of the low-grade lesions. If even a small fraction of the low-grade lesions were to evolve into cancer, then the current screening programmes would be much less efficient and there would be many more interval cancers. Our results represent an indirect argument against the systematic aggressive management of low-grade lesions.

It is usually recommended that only women with a persistent mild dysplasia on cytology should be referred for colposcopy-biopsy (Ellman, 1991). Given the poor sensitivity of smear tests, the cytological follow-up of low-grade lesions is at risk of detecting a false-negative result. Colposcopic studies have already underlined this problem (Jones et al., 1987; Spitzer et al., 1987). Because of its much higher sensitivity for the detection of low-grade lesions, cervicography could be effective for the monitoring of low-grade lesions discovered by cytology among women less than 35 years old. Given that most low-grade lesions occur in young women, follow-up with cervicography would perhaps render safer the 'watch-and-do-nothing' policy, and hence represent an acceptable alternative to systematic colposcopy-biopsy of low-grade lesions.

*High-grade lesions*

In young women, cervicography is more sensitive than smear testing in detecting high-grade lesions, and the high-grade lesions found by cytology are likely to be a subgroup of those found by cervicography. Thus, in women less than 35 years old, a single negative cervicography would perhaps confer higher protection than a single negative smear test. Nevertheless, before envisaging such a strategy, the specificity of cervicography must be further increased. Therefore, as for mammographic screening for breast cancer, the increase in specificity obtained from a double reading of cervicograms has to be evaluated.

*Quality assurance of cytological screening*

The performance of the smear test is closely linked to the quality of sampling and reading. Because of its high sensitivity among young women, cervicography may be used for quality control purposes: for instance, taking cervicograms at the same time as smears would make it possible to detect false-negative cytological results. Indeed, the detection of lesions detected by cervicography but missed by the smear test would allow identification of failures due to sampling or reading problems.

### Conclusions

Our view of cervicography is less optimistic than that of other research teams. However, cervicography may complement smear testing, improving the effectiveness of cervical screening by allowing a more sensitive detection of high-grade lesions in young women, and by avoiding the systematic referral of patients with low-grade lesions for expensive colposcopy-biopsy procedures. In addition, cervicography can be considered as an interesting technique for 'minimal lesion' finding, or for studying the transformation zone status in a large number of women: it could replace colposcopy for this purpose (Rheder & Blythe, 1988). Larger prospective studies are needed to evaluate whether these uses are relevant and economically viable.

This study was supported by a grant from the 'Europe against Cancer' programme of the European Communities (Project No. 506). We thank A. Grivegnée, MD (J. Bordet Institute, Brussels) for his useful comments on a draft of this article.

### References

BRECHT, J.G. & ROBRA, B.P. (1987). A graphic method of estimating the specificity of screening programmes for incomplete follow-up data. *Meth. Inform. Med.*, **26**, 53-59.

COPPLESON, M., MONAGHAN, J.M., MORROW, C.P. & TATTERSALL, M.H.N. (1992). *Gynecologic Oncology: Fundamental Principles and Practice*, 2nd edn. Churchill Livingstone: London.

DAY, N.E. (1989). Screening for cancer of the cervix. *J. Epidemiol. Community Health*, **43**, 103-106.

ELLMAN, R. (1991). Indication for colposcopy from a UK viewpoint. In *Cancer Screening*, Miller, A.B., Chamberlain, J., Day, N.E., Hakama, M. & Prorock, P.C. (eds) pp. 172-183. Cambridge University Press: New York.

IARC (IARC WORKING GROUP ON EVALUATION OF CERVICAL CANCER SCREENING PROGRAMMES) (1986). Screening for squamous cervical cancer: duration of low risk after negative cytology and its implication for screening policies. *Br. Med. J.*, **293**, 659-664.

JONES, D.E.D., CREASMAN, W.T., DOMBROWSKI, R.A., LENTZ, S.S. & WAELTZ, J.L. (1987). Evaluation of the atypical Pap smear. *Am. J. Obstet. Gynecol.*, **157**, 544-549.

LAARA, E., DAY, N.E. & HAKAMA, M. (1987). Trends in mortality from cervical cancer in the nordic countries: association with organized screening programmes. *Lancet*, **i**, 1247-1249.

MILLER, A.B., KN IGHT, J. & NAROD, S. (1991). The natural history of cancer of the cervix, and the implications for screening policy. In *Cancer Screening*, Miller, A.B., Chamberlain, J., Day, N.E., Hakama, M. & Prorock, P.C. (eds) pp. 141-152. Cambridge University Press: New York.

MORRISON, A.S. (1985). *Screening in Chronic Disease*. Oxford University Press: New York.

REHDER, K.E. & BLYTHE, J.G. (1988). Cytology and cervicography compared to cytology alone for human papilloma virus detection. *Colposcopy Gynecol. Laser Surg.*, **4**, 1-7.

SCHATZKIN, A., CONNOR, R., TAYLOR, P. & BUNNAG, B. (1987). Comparing new and old screening tests when a reference procedure cannot be performed on all screenees. *Am. J. Epidemiol.*, **125**, 672-678.

SPITZER, M., KRUMHOLZ, B.A., CHERNYS, A.E., SELTZER, V. & LIGHTMAN, A. (1987). Comparative utility of repeat Papanicolaou smears, cervicography, and colposcopy in the evaluation of atypical Papanicolaou smears. *Obstet. Gynecol.*, **69**, 731-735.

STAFL, A. (1981). Cervicography: a new method for cervical cancer detection. *Am. J. Obst. Gynecol.*, **139**, 815-825.

SZAREWSKI, A., CUZICK, J., EDWARDS, R., BUTLER, B. & SINGER, A. (1991). The use of cervicography in a primary screening service. *Br. J. Obst. Gynaecol.*, **98**, 313-317.

TAWA, K., FORSYTHE, A., COVE, K. & 3 others (1988). A comparison of the Papanicolaou smear and the cervigram: sensitivity, specificity, and cost analysis. *Obstet Gynecol.*, **71**, 229-235.

VERBEEK, A.L.M., VAN DEN BAN, M.C. & HENDRIKS, J.H.C.L. (1991). A proposal for short-term quality control in breast cancer screening. *Br. J. Cancer*, **63**, 261-264.